# Methamphetamine use associated with gun and knife violence: A matched cohort analysis^☆^

**DOI:** 10.1016/j.sopen.2023.04.010

**Published:** 2023-04-27

**Authors:** Areg Grigorian, Matthew Martin, Morgan Schellenberg, Brent Emigh, Jeffry Nahmias, Kazuhide Matsushima, Meghan Lewis, Kenji Inaba

**Affiliations:** a University of California, Irvine, Department of Surgery, Division of Trauma, Burns and Surgical Critical Care, Orange, CA, USA; b University of Southern California, Department of Surgery, Los Angeles, CA, USA; c Warren Alpert Medical School at Brown University, Department of Surgery, Division of Trauma and Critical Care, Providence, RI, USA

**Keywords:** Methamphetamine, Drug use, Violence, Penetrating trauma

## Abstract

**Introduction:**

There may be an association between violence and methamphetamine use. We hypothesized that trauma patients screening positive for methamphetamines are more likely to present after penetrating trauma and have increased mortality.

**Methods:**

The 2017–2019 TQIP was used to 1:2 match methamphetamine (meth**+**) patients to patients testing negative for all drugs (meth**-**). Patients with polysubstance/alcohol use were excluded. Bivariate and logistic regression analyses were performed.

**Results:**

The rate of methamphetamine use was 3.1 %. After matching, there was no difference in vitals, injury severity score, sex, and comorbidities between cohorts (all *p* > 0.05). Compared to meth-, the meth+ group was more commonly sustained penetrating trauma (19.8 % vs. 9.2 %, *p* < 0.001) with stab-wounds being the most common penetrating mechanism (10.5 % vs. 4.5 %, *p* < 0.001). The meth**+** group more commonly underwent surgery immediately from the emergency department (ED) (20.3 % vs. 13.3 %, p < 0.001). The associated risk of death in the ED was higher for the meth**+** group (OR 2.77, CI 1.45–5.28, *p* = 0.002), however, the risk was similar for patients that were admitted or received an operation (*p* = 0.065).

**Conclusion:**

Trauma patients using methamphetamine more commonly presented after gun or knife violence and required immediate surgical intervention. They also have increased associated risk of death in the ED. Given these serious findings, a multidisciplinary approach in helping curtail the worsening epidemic of methamphetamine use appears warranted as it is related to penetrating trauma and outcomes.

**Level of evidence:**

IV.

## Introduction

Methamphetamines, known by multiple street-names including “speed”, “meth” and “ice” are an incredibly powerful and addictive stimulant producing hyperawareness, hallucinations, paranoia, and euphoria [1]. According to a 2020 national survey, the estimated rate of past year methamphetamine use among adults has increased by 20 % from 1.6 million in 2015 to >2 million in 2019 [2]. Methamphetamine use has reached epidemic proportions and is an expanding public health issue which has been demonstrated to significantly increase hospital length of stay (LOS), complications, and mortality [3].

Hospitalizations related to methamphetamine use have increased to a greater degree than hospitalizations associated with other illicit substances and the annual hospital costs related to amphetamine-use has risen significantly to over $2 billion annually [4]. Much of this burden has been placed on trauma systems and providers as a lot of these hospitalizations are related to injury [5]. Furthermore, single-center studies have demonstrated that trauma patients using methamphetamines are more likely to present after violent or illegal activities [6,7]. As such, trauma centers are in a unique position to identify and develop systematic approaches to help curtail the negative consequences of illicit drug use in our communities.

The aim of this study was to identify the current national rate of methamphetamine use in trauma patients and determine its effect on clinical outcomes hypothesizing an increased risk of early and overall death, and complications for patients screening positive for methamphetamines. We additionally hypothesized that trauma patients with a positive methamphetamine screen are more likely to present after penetrating trauma.

## Methods

This study was deemed exempt by our institutional review board as it utilizes a national deidentified database. The 2017–2019 TQIP database was queried for patients 18-years of age and older that received an initial drug screen upon presentation. We did not use earlier years of the TQIP database as prior to 2017, patients were coded with both ICD-9 and 10 diagnosis/procedure codes. Starting in 2017, all patients were coded with ICD-10 codes. Since there may be a “vices-paradox” with other illicit drugs and/or alcohol in trauma patients, we excluded patients with a positive alcohol screen or testing positive for any other potentially illicit drugs (cocaine, barbiturates, cannabinoids, methadone, opioid, oxycodone, phencyclidine, and tricyclic antidepressants) [[8], [9], [10]]. We aimed to compare two groups: trauma patients screening positive for methamphetamine (meth+) on admission to trauma patients screening negative for all other drugs and alcohol (meth-).

Due to the observed imbalance of patients in both groups we performed a 1:2 propensity score-matched analysis. Matched variables included patient age, sex, heart rate > 120 beats per minute on arrival, systolic blood pressure < 90 mmHg on arrival, respiratory rate > 22/min on arrival, injury severity score, and comorbidities including anticoagulant use, congestive heart failure, chronic kidney disease, cerebrovascular accident, chronic obstructive pulmonary disease, diabetes, functional dependance, hypertension, myocardial infarction, and current smoker. We included in our analysis only those cases that were within 0.001 of the estimated logit [11].

The primary outcomes were early death in the emergency department (ED) and overall mortality. The secondary outcomes included trauma mechanisms and in-hospital complications. Complications measured included acute respiratory distress syndrome (ARDS), cardiac arrest, deep vein thrombosis (DVT), pulmonary embolism (PE), unplanned intubation, acute kidney injury (AKI), myocardial infarction (MI), unplanned return to the operating room, unplanned intensive care unit (ICU) admission, and pneumonia. Other measured outcomes included the severity of injury (defined by abbreviated injury scale [AIS]), total hospital length of stay (LOS), ICU LOS, and ventilator days.

Bivariate analyses were performed using a Mann-Whitney-*U* test to compare continuous variables and chi-square to compare categorical variables for bivariate analysis. Categorical data was reported as percentages, and continuous data was reported as medians with interquartile range. All *p*-values were two-sided, with a statistical significance level of <0.05. All analyses were performed with IBM SPSS Statistics for Windows (Version 28, IBM Corp., Armonk, NY).

## Results

From 83,101 patients, 2578 (3.1 %) screened positive for methamphetamines. Using a 1:2 propensity-score model, 1613 meth+ patients were matched to 3226 meth- patients. There was no difference in age, sex, comorbidities, injury severity, or vitals on admission between the two groups (all *p* > 0.05). The average median age for meth+ was 40-years and the two most common comorbidities were smoking (38.4 %) and hypertension (12.6 %) (Table 1).

**Table 1:** Demographics for 1:2 matched meth+ and meth- trauma patients.

	Meth-	Meth+	
Characteristic	(*n* = 3226)	(*n* = 1613)	p-Value
Age, year, median (IQR)	40 (32, 52)	40 (32, 52)	0.98
Male, n (%)	2444 (75.8 %)	1222 (75.8 %)	1.00
Comorbidities, n (%)			
Anticoagulant use	12 (0.4 %)	6 (0.4 %)	1.00
CHF	2 (0.1 %)	1 (0.1 %)	1.00
Chronic kidney disease	0	0	–
Cerebrovascular accident	2 (0.1 %)	1 (0.1 %)	1.00
COPD	24 (0.7 %)	12 (0.7 %)	1.00
Diabetes	120 (3.7 %)	60 (3.7 %)	1.00
Functionally dependent	2 (0.1 %)	1 (0.1 %)	1.00
Hypertension	406 (12.6 %)	203 (12.6 %)	1.00
Myocardial infarction	0	0	–
Smoker	1238 (38.4 %)	619 (38.4 %)	1.00
Vitals on admission, n (%)			
Hypotensive (SBP < 90 mmHg)	16 (0.5 %)	8 (0.5 %)	1.00
Tachycardia (HR > 120/min)	174 (5.4 %)	87 (5.4 %)	1.00
Tachypnea (RR > 22/min)	628 (19.5 %)	314 (19.5 %)	1.00

CHF = congestive heart failure; COPD = chronic obstructive pulmonary disease, SBP = systolic blood pressure; HR = heart rate; RR = respiratory rate.

Compared to meth-, patients that were meth+ had a similar median ISS (9, *p* = 0.98) and injury severity (AIS > 2) for the head (15.1 % vs. 15.7 %), spine (4.7 % vs. 4.9 %), thorax (16.2 % vs. 17.9 %), and abdomen (5.5 % vs. 5.0 %) (all *p* > 0.05). However, meth+ patients more commonly presented after a stab (10.5 % vs. 4.5 %, *p* < 0.001) or gunshot wound (8.3 % vs. 4.6 %, p < 0.001) (Table 2).

**Table 2:** Injury patterns for 1:2 matched meth+ and meth- trauma patients.

	Meth-	Meth+	
Characteristic	(n = 3226)	(n = 1613)	p-Value
ISS, median (IQR)	9 (5, 14)	9 (5, 14)	0.98
Blunt mechanism, n (%)			
Pedestrian struck	146 (4.5 %)	120 (7.4 %)	<0.001
Bicycle	105 (3.3 %)	77 (4.8 %)	0.009
Motorcycle accident	382 (11.8 %)	158 (9.8 %)	0.04
Motor vehicle crash	1158 (35.9 %)	465 (28.8 %)	<0.001
Fall	735 (22.8 %)	260 (16.1 %)	<0.001
Penetrating mechanism, n (%)			
Knife wound	145 (4.5 %)	169 (10.5 %)	<0.001
Gun wound	147 (4.6 %)	134 (8.3 %)	<0.001
AIS (grade ≥ 2), n (%)			
Head	505 (15.7 %)	244 (15.1 %)	0.64
Spine	157 (4.9 %)	76 (4.7 %)	0.81
Thorax	579 (17.9 %)	261 (16.2 %)	0.13
Abdomen	161 (5.0 %)	88 (5.5 %)	0.49

ISS = injury severity score; AIS = abbreviated injury scale.

Patients in the meth+ group were more likely to require ICU admission (38.4 % vs. 31.1 %, *p* < 0.001), ventilator need (18.6 % vs. 11.3 %, p < 0.001) and an immediate operation from the emergency department (20.3 % vs. 13.3 %, p < 0.001), compared to the meth- group. The meth+ group was also more likely to develop an in-hospital DVT (1.2 % vs. 0.7 %, *p* = 0.04) and undergo an unplanned return to the OR (1.5 % vs. 0.5 %, *p* = 0.001). The rate of death in the ED was higher for meth+ patients (0.9 % vs. 0.3 %, *p* = 0.005) but the overall mortality rate was similar (2.8 % vs. 3.4 %, *p* = 0.28) (Table 3).

**Table 3:** Clinical outcomes for 1:2 matched meth+ and meth- trauma patients.

	Meth-	Meth+	
Characteristic	(n = 3226)	(n = 1613)	p-Value
ICU admission, n (%)	1002 (31.1 %)	620 (38.4 %)	<0.001
Ventilator need, n (%)	365 (11.3 %)	300 (18.6 %)	<0.001
Emergent/urgent operation, n (%)	429 (13.3 %)	327 (20.3 %)	<0.001
Complications, n (%)			
Acute respiratory distress syndrome	7 (0.2 %)	3 (0.2 %)	0.82
Cardiac arrest	10 (0.3 %)	5 (0.3 %)	1.00
Deep vein thrombosis	22 (0.7 %)	20 (1.2 %)	0.04
Pulmonary embolism	17 (0.5 %)	8 (0.5 %)	0.89
Unplanned intubation	29 (0.9 %)	17 (1.1 %)	0.60
Acute kidney injury	9 (0.3 %)	15 (0.9 %)	0.002
Myocardial infarction	0	4 (0.2 %)	0.005
Unplanned return to OR	17 (0.5 %)	21 (1.5 %)	0.001
Unplanned ICU admission	38 (1.2 %)	19 (1.2 %)	0.99
VAP	12 (0.4 %	12 (0.7 %)	0.08
LOS, days, median (IQR)	4 (2, 6)	4 (2, 8)	<0.001
ICU LOS, days, median (IQR)	3 (2, 5)	3 (2, 6)	0.32
Ventilator, days, median (IQR)	3 (2,7)	3 (2, 6)	0.70
Mortality, n (%)			
Death in ED	9 (0.3 %)	14 (0.9 %)	0.005
Death after admission from ED	109 (3.4 %)	45 (2.8 %)	0.276

ICU = intensive care unit; VAP = ventilator-associated pneumonia; LOS = length of stay; ED = emergency department.

## Discussion

Methamphetamines are an increasingly popular medication of use for multiple reasons including the relatively low acquisition cost and ease of access (e.g., street and pharmaceutical versions). This national analysis identified a correlation between methamphetamine use and penetrating trauma mechanisms which are often associated with violent behavior. Furthermore, although meth+ and meth- patients present with similar severity of injuries and initial vitals, meth+ patients more often required ICU admission, mechanical ventilation, and immediate operation. Although the overall mortality rate was similar between the two groups, the rate of early death was higher for meth+ patients suggesting a more vulnerable physiologic baseline.

Methamphetamine use can result in violent behavior. This relationship may be dose-dependent and independent of the violence risk associated with psychotic symptoms [12]. These changes are primarily mediated through molecular changes in the dopamine system, and both acute and chronic effects can permanently alter nerve terminal pathways resulting in paranoia, psychosis, anxiety, impaired motor skills, and rapid cognitive decline [13,14]. The resulting impaired cognition may be fueled by violent outbursts ultimately leading to aggression, and may place users at risk for injuries with a knife or firearm [15], as was identified in this national retrospective study. In addition, prior reports have demonstrated that patients screening positive for methamphetamines may have an increased hospital LOS, even in the minimally injured population [3,16]. Our study confirms these previous reports and suggests these patients utilize crucial resources such as ICU beds more often than similarly matched non-methamphetamine users.

Although methamphetamine use can lead to aggression, it may also place users in vulnerable situations where they may be the victim of a violent attack. Although our study demonstrates a positive relationship between methamphetamine use and penetrating trauma mechanisms, we are unable to determine if the patient is a victim of the trauma or the perpetrator. Methamphetamine use can disrupt the frontostriatal regions involved in cognitive functions including verbal memory, psychomotor function and the ability to manipulate information [17,18]. This can result in increased cognitive reaction times and may reflect an impaired ability to suppress irrelevant information making the user appear distractable and have difficulty concentrating [19]. Users may then exercise poor judgement placing them in high-risk situations where they may be victimized using knife or firearm violence.

Methamphetamine use can result in a poorer physiologic state making it more challenging to tolerate a “second-hit” from a traumatic injury. Methamphetamine use is clearly associated with cardiovascular disease resulting in atherosclerotic plaque, vasospasms, morphologic changes in coronary and peripheral vessels, and electrical remodeling of cardiac tissue resulting in potentially fatal arrythmias [20]. Furthermore, over 10 % of methamphetamine-positive patients presenting to the hospital have evidence of AKI [21]. This may be related to systemic vasoconstriction, volume depletion, labile blood pressures, hyperthermia, and rhabdomyolysis [22,23]. Our study also found a higher rate of AKI in trauma patients screening positive for methamphetamines. Furthermore, methamphetamines can disrupt immune-related signal pathways disrupting the response to injury [24]. Although the meth+ patients in our study had similar vitals on admission and severity of injury to their matched meth- counterparts, they clearly had a higher rate of early decompensation requiring a higher rate of ICU admission, early operative intervention, ventilator need and early death. This suggests that methamphetamine use makes users less tolerable of similar traumatic injuries compared to their meth- counterparts. Future research is needed to confirm these findings and elucidate whether pharmacologic interventions (e.g., benzodiazepines or beta blockers) typically used to treat symptoms of methamphetamines may help mitigate physiologic derangements and improve outcomes.

One potential approach to addressing the problem of methamphetamine use among trauma patients is the implementation of a multidisciplinary team consisting of trauma surgeons, emergency physicians, mental health professionals, and addiction specialists. This team can work together to develop a comprehensive care plan for patients who screen positive for methamphetamine use. This care plan could include early identification and intervention, referral to addiction treatment, collaboration with community-based organizations and post-discharge follow-up and support. By implementing these strategies, trauma providers can play a critical role in addressing the complex issue of methamphetamine use among trauma patients and ultimately contribute to improved patient outcomes and a reduction in the burden of methamphetamine-related hospitalizations on the healthcare system.

Limitations of this study include those inherent to large database studies, such as selection and reporting bias. There is also a lack of granular information in the database regarding methamphetamine use. Specifically, the TQIP database does not provide the amount of methamphetamine used, chronicity of use, route of administration, or even the temporal relationship of methamphetamine use and the traumatic injuries. Additionally, there is no standardized approach to methamphetamine testing across trauma centers. Another major limitation is the inability to identify if the trauma patient was the victim or perpetrator in penetrating trauma mechanisms. The TQIP database does not include information on determination of drug intervention, drug rehabilitation following discharge, or the rate of recidivism. And finally, we are unable to make any claims of causation due to the retrospective nature of the study.

## Conclusions

Trauma patients positive for methamphetamine more commonly presented after gun or knife violence and more often underwent immediate surgical intervention compared to patients negative for all drugs and alcohol. Methamphetamine positive patients also had a higher rate of ICU admission, ventilator need and increased associated risk of death in the ED. The physiologic reasons for this important association warrants further investigation. Given these serious findings, a multidisciplinary approach in helping curtail the worsening epidemic of methamphetamine use appears warranted as it is related to penetrating trauma and outcomes.

## CRediT authorship contribution statement

All authors contributed to the study conception and design. Data collection and statistics were performed by AG. All authors participated in data analysis. The first draft of the manuscript was written by AG and all authors commented on previous versions of the manuscript. All authors read and approved the final manuscript.

## Funding source

This research did not receive any specific grant from funding agencies in the public, commercial, or not-for-profit sectors.

## Ethics approval

This research used a deidentified national database and so a waiver was issued from our local institutional review board.

## Declaration of competing interest

The authors report no conflicts of interest.
